# Cardioprotective effects of the ranolazine in myocardial infarction mediated by stimulation of the endogenous mediators involved in ischemic preconditioning

**DOI:** 10.1590/acb410926

**Published:** 2026-03-20

**Authors:** Junaid Tantray, Francisco Sandro Menezes-Rodrigues, José Mario Podanosque, Fernando Sabia Tallo, Afonso Caricati-Neto, Ashish Kumar Sharma, Akhilesh Patel, Rajesh Kumar Sharma, Shivam Singh, Bolatkan Arlan Beibituly

**Affiliations:** 1 Nims University - Nims Institute of Pharmacy - Department of Pharmacology - Jaipur, Rajasthan, India.; 2 Universidade Federal de São Paulo - Postgraduate Program in Cardiology - São Paulo (SP), Brazil.; 3 Universidade Federal de São Paulo - Postgraduate Program in Interdisciplinary Surgical Science - São Paulo (SP), Brazil.; 4 Universidade Federal de São Paulo - Department of Pharmacology - São Paulo (SP), Brazil.; 5 Shree Guru Gobind Singh Tricentenary University - College of Pharmacy - Gurgaon, Haryana, India.; 6 KAAF University - Faculty of Health and Allied Science - Department of Pharmacy - Accra, Ghana.; 7 Ministry of Health of the Republic of Kazakhstan - National Scientific Center of Phthisiopulmonology - Interventional Cardiology - Almaty, Republic of Kazakhstan.

**Keywords:** Ranolazine, Ischemic Preconditioning, Nitric Oxide, Adenosine, Bradykinin

## Abstract

**Purpose:**

Hypothesis centered on the idea that ranolazine could induce responses similar to ischemic preconditioning, involving nitric oxide, adenosine, bradykinin, and adenosine triphosphate (ATP)-dependent potassium channels.

**Methods:**

Ischemia-reperfusion injury was established using Langendroff technique. Thirty-minute ischemia and 120-minute reperfusion to coronary artery to isolated heart were model of myocardial infarction. There were studied the following groups: control (ischemia-reperfusion), ischemic preconditioning, ranolazine (10 µmol/L), ranolazine + L-NAME (30 µmol/L) and ranolazine + aminoguanidine (30 µmol/L), ranolazine + theophylline (50 µmol/L), ranolazine + aminophylline (50 µmol/L), ranolazine + icatibantl (100 µmol/L), ranolazine + bromelain (250 µmol/L), ranolazine + 5-hydroxydecanoate (30 µmol/L), and ranolazine + glimepiride (50 µmol/L) in perfusate.

**Results:**

Ranolazine and ischemic preconditioning groups demonstrated cardioprotective effects by reducing infarct size, as well as levels of lactate dehydrogenase (LDH), creatine phosphokinase myoglobin (CK-MB), and troponin I and cardiac parameters like left ventricular developed pressure (LVDP) and left ventricular maximum rate of contraction (dP/dT_max_). Maximum rate of relaxation (dP/dT_min_) was improved. Conversely, treatments with L-NAME, aminoguanidine, theophylline, aminophylline, icatibant, bromelain, 5-hydroxydecanoate, and glimepiride increased infarct size, LDH, CK-MB, and troponin I levels, and cardiac parameters like LVDP, dP/dT_max_, and dP/dT_min_ were depressed. This data provides evidence that ranolazine employs nitric oxide, adenosine, bradykinin, and ATP-dependent potassium channels as secondary messengers in cardioprotection.

**Conclusion:**

ranolazine can be a pharmacological alternative to surgical ischemic preconditioning utilized prior to interventional procedures like coronary artery bypass graft surgery and heart transplantation, offering improved patient compliance.

## Introduction

Estimates suggest that cardiovascular diseases claim the lives of approximately 17.5 million individuals annually, constituting around one-third of all global deaths. Over the past century, significant advancements have been made in cardiovascular sciences, spanning from non-invasive diagnostic methods to invasive interventions such as coronary artery bypass graft surgery and heart transplantation. Despite these advancements, cardiovascular diseases persist as the primary cause of morbidity and mortality worldwide, imposing a substantial financial burden on healthcare systems. The preferred approach for managing acute coronary syndrome is myocardial reperfusion, but the sudden reintroduction of blood flow exacerbates tissue damage caused by prior ischemia, a phenomenon known as ischemia/reperfusion injury (IRI). One proposed theory to explain this phenomenon revolves around the reperfusion-induced imbalance between vasodilator and vasoconstrictor chemicals^1^.

The investigation of a brand-new cardio protection concept has been sparked by efforts to develop ways to prevent and treat this injury. The prevention of permanent myocardial injury and the protection of ventricular dysfunction, which results in heart failure, are the current hot topics of research and discussion. The term “cardio protection” generally refers to all procedures and techniques that lessen or even stop myocardial deterioration and so help to preserve the heart^2^.

Regrettably, most findings from animal studies have not been reproducible in human contexts, likely due to inherent differences in risk factors and comorbidities^3,4^.

Over the past few decades, extensive research has unveiled that cardiac cells possess various defense mechanisms aimed at mitigating the damage caused by ischemia/reperfusion. These mechanisms offer protection not only in the immediate aftermath of acute coronary syndrome but also mitigate the long-term impacts of myocardial infarction, extending beyond the myocardium to areas proximal and distal to the heart. Examples of such protection include pre-conditioning and post-conditioning of myocardial cells against ischemia/reperfusion events. Despite their significance, the precise cellular mechanisms governing these phenomena remain elusive, although they are likely intricate and may involve factors originating from extracardiac sources^5^.

When initial antianginal therapies fail to adequately manage angina symptoms in symptomatic patients, ranolazine is employed as an additional treatment. Ranolazine functions by blocking sodium voltage-dependent channels, suggesting its potential role in mitigating the reperfusion process by preventing the accumulation of excessive sodium and calcium during ischemia. In this study, isolated albino Wistar rats underwent a simulated ischemia and reperfusion protocol to evaluate the cardioprotective effects of ranolazine^6^. The most recent advancements in the field of cardioprotection have been assessed through a review of state-of-the-art literature^7-12^.

Initially approved by the Food and Drug Administration (FDA) in 2006, ranolazine, a piperazine derivative, offers a unique mechanism of action for managing symptoms in patients with chronic angina. Through its pharmacological properties, it enhances the heart’s utilization of oxygen by blocking the late sodium current (INaL) in cardiomyocytes and redirecting fatty acid oxidation towards glucose oxidation. Despite its efficacy, the precise mechanism by which ranolazine operates remains incompletely understood^13,14^. It is known to exert its therapeutic effects by inhibiting the influx of sodium into cardiac cells via sodium channels, which under pathological conditions may fail to close after becoming inactivated^15^. Additionally, there is a suggestion that ranolazine might reduce calcium accumulation in cardiac cells during ischemia by blocking the INaL current^16^.

Clinical evidence strongly indicates that both ranolazine and ischemic preconditioning confer cardioprotection. Our research aimed to investigate whether the cardioprotective effects induced by ranolazine mirror those elicited by ischemic preconditioning. If our hypothesis is validated, ranolazine could potentially serve as a pharmacological alternative to the surgical procedure of ischemic preconditioning prior to interventional techniques such as coronary artery bypass graft surgery and heart transplantation.

## Methods

### Animals

The Animal House at NIMS University, in Rajasthan, Jaipur, India, provided the Wistar albino rats used in this investigation. We selected rats weighing between 180 and 200 grams, regardless of their sex, for this study. We provided them with *ad libitum* access to tap water and a standard laboratory rat diet. The experimental protocol was ethically approved by the Institutional Animal Ethics Committee (2022) at NIMS University, under registration number 1203/PO/Re/S/09/CPCSEA, in compliance with the guidelines set forth by the Committee for Control and Supervision of Experiments on Animals (CCSEA), Government of India, New Delhi, India.

Above mentioned ethical approval are as per standard guidelines of the CCSEA is a statutory Committee of Department of Animal Husbandry and Dairying, Ministry of Fisheries, Animal Husbandry and Dairying, constituted under the Prevention of Cruelty to Animals Act, 1960. CCSEA is duty bound to take all such measures as necessary to ensure that animals are not subjected to unnecessary pain or suffering before, during or after performance of experiments on them. For this purpose, the committee formulated the Breeding of and Experiments on Animals (Control & Supervision) Rules, 1998 (amended in 2001 and 2006), to regulate the experimentation on animals. Under the provisions of the above rules, establishments who are engaged in bio-medical research, breeding and trading of laboratory animals are required to get themselves registered with CCSEA. There are 19 members in the present CCSEA, wherein Dr. Abhijit Mitra, Animal Husbandry commissioner, is the chairman of CCSEA, and Dr. S. K. Dutta, Joint commissioner (Animal Welfare), is the member secretary of CCSEA.

### Drugs and chemicals

The drugs and chemicals used during experimental procedure are mentioned in Table 1.

**Table 1:** Drugs and chemicals and their procurement.

Drug	Procurement
Ranolazine	Glenmark Pharmaceuticals Limited, Maharashtra
Ketamine	Troika Pharmaceuticals Limited, Uttarakhand, India
L-NAME (Nώ-nitro-L-arginine methyl ester)	Tokyo Chemical Industry Co. Ltd., Toshima, Kita-Ku., Tokyo, Japan
Aminoguanidine	BLD Pharmatech (India) Pvt. Ltd., Hyderabad, Telangana, India
Theophylline	Zydus Healthcare Limited, Hyderabad, India
Aminophylline	Rathi Laboratories, Patna, India
Icatibant injection 30 mg/3 mL (10 mg/mL)	Cipla Ltd. 550 S. Research Place Central Islip New York, United States of America
Bromelain powder	CSV Pharmaceuticals. Registered Office: Plot no A 203, Sector 69, Noida, Uttar Pradesh, 201301, India Factory Address: Mauza Sidcul Industrial Area Sidcul, Dehradun, Uttarakhand, India
5-hydroxydeconate	BLD Pharmatech Limited, Telegana, India
Glimepride	Medely Pharmaceuticals Ltd., Mumbai, India
Heparin	BE Pharmaceuticals Private Limited, Telangana, India
Triphenyl tetrazolium chloride	BLD Pharmatech Private Limited, Telangana, India
NaHCO_3_, NaH_2_PO_4_	Qualikems Fine Chem Private Limited, Vadodara, India
MgSO_4_, CaCl_2_, diethyl ether, carboxymethylcellulose	Central Drug Store (P) Limited, New Delhi, India
KCl, dextrose, NaCl	Ases Chemicals, Jodhpur, India
Na_2_HPO_4_	RFCL Limited, New Delhi, India

Source: Elaborated by the authors.

### Infarct size measurement

**Figure 1:**
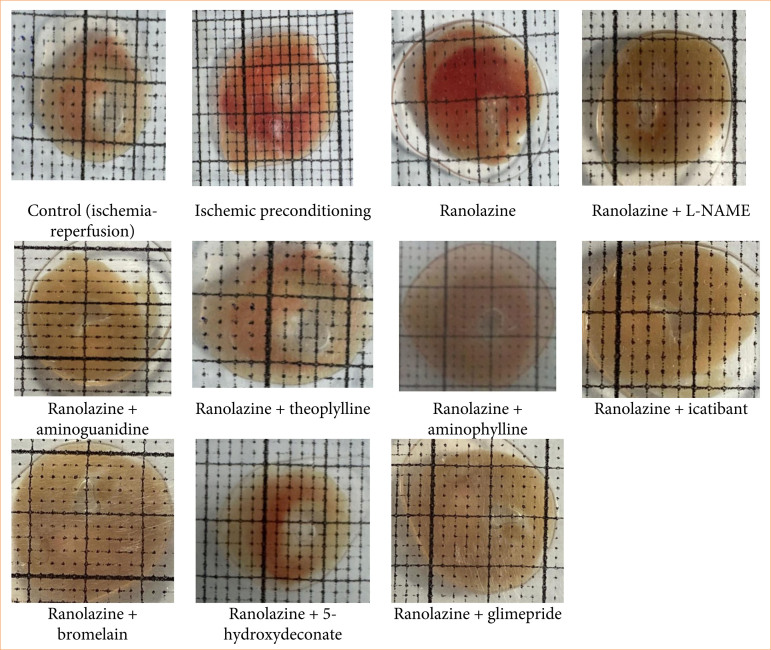
Representative photos of different groups of infarct size by volume method.

Infarct size measurement was carried by volume method (Fig. 1)^17-19^.

*The heart slices were stained with the incubation in 1% triphenyltetrazolium chloride buffer at 37°C in 0.2 M Tris buffer (pH 7.4) for 10 minutes. Source: Elaborated by the authors.

### Surgical preparations

Apparatus: Langendorff apparatus (INCO, Ambala, India); electrocardiography (BPL CARDIAART 108TDIGI, New Delhi, India) and pressure transducer (AD Instruments, Australia) to record left ventricular developed pressure (LVDP) and its first derivatives left ventricular maximum rate of contraction (dP/dT_max_) and maximum rate of relaxation (dP/dT_min_).

*Ex-vivo* pharmacological and physiological investigations in animals use the Langendorff cardiac test, also known as the isolated perfused cardiac test. Named after the German physiologist Oskar Langendorff, this technique enables the examination of cardiac muscle contractile force and heart rate without the complexities encountered in intact organisms. In the Langendorff setup, the heart is extracted from the animal by severing the blood vessels, followed by retrograde perfusion through the aorta using a nutrient-rich, oxygenated solution such as Krebs-Henseleit solution^17-19^.

### Procedure

Before opening the chest, Wistar albino rats (180-200 grams of either sex) were anesthetized with ketamine (100 mg/kg, i.p.) and administered heparin (500 IU, i.p.) approximately 15 minutes before the animals’ sacrifice. After pericardium removal, we swiftly excised the heart and mounted it on the Langendorff apparatus and initiated retrograde perfusion of the aorta with Krebs-Henseleit buffer solution at a constant pressure of 70 mmHg, oxygenating it with 95% O_2_ and 5% CO_2_. Sacrifice of the animal has done by overdose of anesthesia and disposed of by biomedical waste of the university. Flow rate was maintained at 6-8 mL/min. The heart was enclosed in a double wall jacket, the temperature of which was maintained by circulating water at 37°C. A fluid filled latex balloon was inserted into left ventricle and was connected to a pressure transducer (AD Instruments, Australia) to record LVDP and its first derivatives dp/dt_max_ and dp/dt_min_. Ischemia was induced for 30 minutes by ligating the left anterior descending coronary artery, followed by 120 minutes of reperfusion.

**Figure 2:**
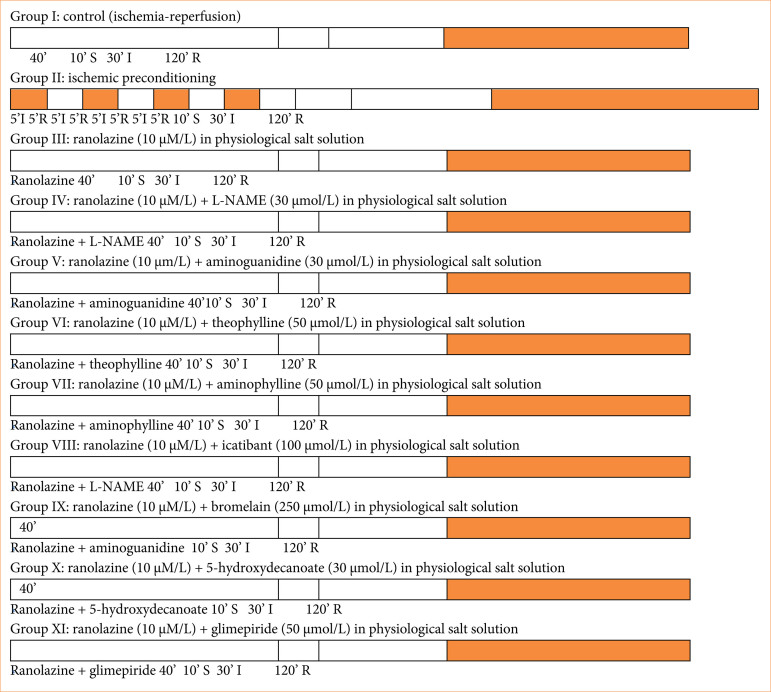
Diagrammatic representation of the experimental protocol.

Electrocardiography was performed using silver electrodes attached to specific locations on the heart at various time points throughout the experiment. Coronary effusion was collected at the end of reperfusion (120-minutes reperfusion) for the determination of lactate dehydrogenase (LDH), creatine phosphokinase myoglobin (CK-MB) and troponin I levels. After the IRI protocol, we removed the hearts from Langendorff apparatus. Hearts were frozen in deep freeze for 24 h at -20°C. After 24 hours, heart was taken out of deep freeze, and 1-mm thickness slices were made. The heart slices were incubated in 1% triphenyltetrazolium chloride (TTC) buffer at 37°C in 0.2 M Tris buffer (pH 7.4) for 10 minutes. After incubation, the slices were taken out, and the infarct size was measured following a saline wash. Infarct size was quantified using the volume method and expressed as a percentage of the total myocardial volume. This protocol included pre-treatment with drugs in Krebs-Henseleit buffer physiological salt solution, before inducing IRI to investigate their mechanism of action as per protocol (Fig. 2)^17-19^.

I: ischemia at minutes-time interval; R: reperfusion at minutes-time interval; S: stabilization at minutes-time interval.Source: Elaborated by the authors.

### Experimental protocol

There were 66 Wistar albino rats (either sex) utilized in this investigation, divided into 11 groups, with an average of six animals per group (Table 2, Fig. 2).

**Table 2:** Different type of groups with number of animals*.

Group number	Groups description	Number of animals
I	Control (ischemia-reperfusion)	6
II	Ischemic preconditioning	6
III	Ranolazine (10 µmol/L)	6
IV	Ranolazine (10 µmol/L) and L-NAME (30 µmol/L)	6
V	Ranolazine (10 µmol/L) and aminoguanidine (30 µmol/L)	6
VI	Ranolazine (10 µmol/L) and theoplylline (50 µmol/L)	6
VII	Ranolazine (10 µmol/L) and aminoplylline (50 µmol/L)	6
VII	Ranolazine (10 µmol/L) and icatibant (100 µmol/L)	6
IX	Ranolazine (10 µmol/L) and bromelain (250 µmol/L)	6
X	Ranolazine (10 µmol/L) and 5-hydroxydecanoate (30 µmol/L)	6
XI	Ranolazine (10 µmol/L) and glimepiride (30 µmol/L)	6

* The concentrations of drugs in Krebs-Henseleit buffer physiological salt solution are mentioned in the article.

Source: Elaborated by the authors.

### Staining procedure

After ischemia-reperfusion protocol, the hearts were removed from the Langendorff apparatus and placed in a refrigerator for 24 hours at -20°C. Subsequently, 1-mm slices of the frozen heart were prepared. These heart slices were then stained with 1% TTC buffer by incubating in biochemical oxygen demand incubator for 10 minutes. After incubation, the slices were taken out, and the infarct size was measured following a saline wash^17-19^.

### Estimation of lactate dehydrogenase, CK-MB, and cardiac troponin I in coronary effluent

#### Lactate dehydrogenase assay

LDH was quantified in the coronary fluid utilizing the integrated VITROUS system 5600 apparatus. The underlying principle involves LDH catalyzing the conversion of lactate and nicotinamide adenine dinucleotide (NAD) to pyruvate and reduced nicotinamide adenine dinucleotide (NADH). The resulting pyruvate reacts with 2,4-DNPH to form the corresponding hydrazone, producing a brown color in an alkaline environment. The intensity of this color is directly proportional to the LDH activity and is assessed spectrophotometrically at a wavelength of 440 nm.

#### Creatine phosphokinase myoglobin assay

The concentration of creatine phosphokinase myoglobin (CK-MB) in the coronary fluid was also assessed using the VITROUS system 5600 apparatus. The principle involves creatine phosphokinase catalyzing the conversion of creatine phosphate and ADP to creatine and adenosine triphosphate (ATP). At a pH of 7.4, CK-MB promotes the forward reaction. The resulting creatine reacts with diacetyl and naphthol in an alkaline environment, producing a pink color. The intensity of this color is directly proportional to the enzymatic activity and is measured spectrophotometrically at a wavelength of 520 nm. Activators such as Mg2 + and cysteine are added, while p-chloromercuric benzoate is introduced to halt the reaction by inactivating the enzyme.

### Cardiac troponin I assay

Rat cardiac troponin I kit provided by Abcam-Allied Scientific is a single-wash 90-minute simple step used to quantify rat cardiac troponin I with a sensitivity of 7.7 pg/mL. The assay uses a simple mix-wash-read protocol with just one incubation and one wash step.

**Figure 3:**
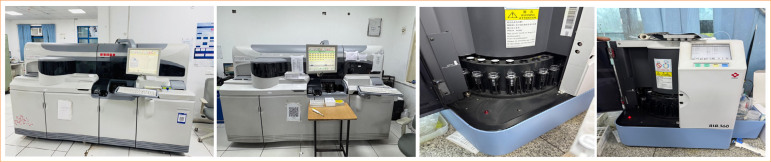
VITROS 5600 Integrated system, Ortho-Clinical Diagnostics, United States of America.

Source: Elaborated by the authors.

The coronary effluent samples are tested for cardiac biomarkers like LDH, CK-MB, and troponin I in VITROS 5600 Integrated system, Ortho-Clinical Diagnostics, United States of America (Fig. 3), whose specifications and photographs are attached as Suppl. Mat. (https://www.quidelortho.com/us/en/products/vitros-systems/vitros-5600-integrated-system-analyzer).

### Statistical analysis

Enzymatic data and infarct size values were presented as mean ± standard error of the mean. Statistical significance was assessed using one-way analysis of variance. Tukey’s multiple comparison test was utilized as a *post hoc* test for comparisons with the control group, while Student’s t-test was employed for multiple comparisons between groups. A significance level of *p* < 0.05 was considered statistically significant. Statistical analysis was conducted using SigmaPlot v15 software.

## Results

### Effect of control (ischemia-reperfusion) and ischemic preconditioning group on infarct size, LDH, CK-MB, troponin I, LVDP, dP/dT_max_, and dP/dT_min_

**Figure 4:**
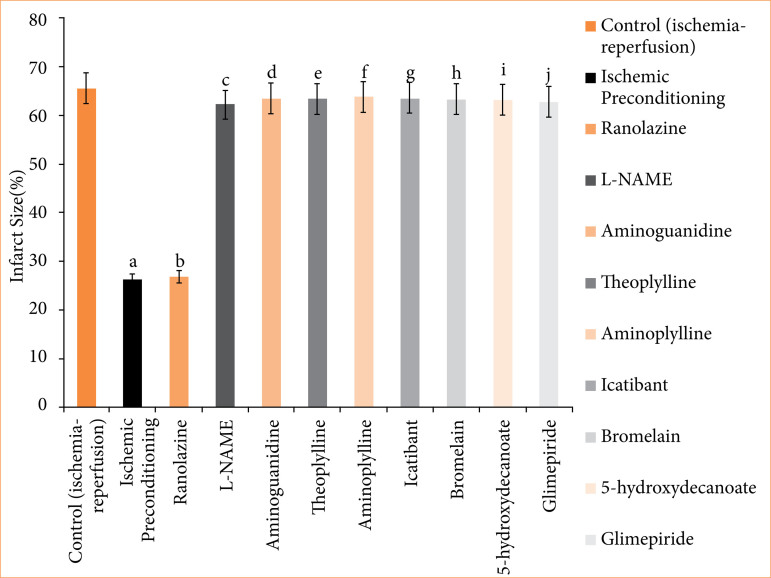
Infarct size of rat’s heart. The values presented are the mean ± standard error of the mean of 11 experimental groups, with six in each experimental group. Data were statistically analyzed by using one-way analysis of variance followed by Tukey’s multiple comparisons range test. In terms of significance, p > 0.05 indicates a non-significant value, *p ≤ 0.05 denotes a significant value, **p < 0.01 denotes a very significant value, and ***p < 0.001 denotes an extremely significant value. Notations such as c, d, e, f, g, h, i, and j indicate comparisons between ranolazine alone group (p < 0.05), in which a comparison marked with a and b letter denotes statistical significance verses control (ischemia-reperfusion) (p < 0.05). All combined treatments show significant differences from ranolazine alone (p < 0.05) and are not different from the control (p > 0.05).

Source: Elaborated by the authors.

**Figure 5:**
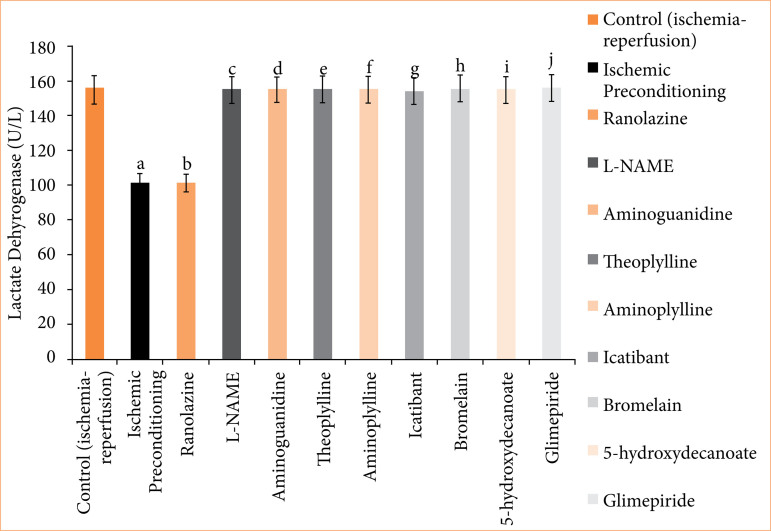
Lactate dehydrogenase (LDH) release in coronary effluent of isolated rat heart at 120-minute reperfusion. The values presented are the mean ± standard error of the mean of 11 experimental groups, with six in each experimental group. Data were statistically analyzed by using one-way analysis of variance followed by Tukey’s multiple comparisons range test. In the context of significance, p > 0.05 indicates a non-significant value, *p ≤ 0.05 denotes a significant value, **p < 0.01 denotes a very significant value, and ***p < 0.001 denotes an extremely significant value. Notations such as c, d, e, f, g, h, i, and j indicate comparisons between ranolazine alone group (p < 0.05), in which a comparison marked with a and b letter denotes statistical significance verses control (ischemia-reperfusion) (p < 0.05). All combined treatments show significant differences from ranolazine alone (p < 0.05) and are not different from the control (p > 0.05).

Source: Elaborated by the authors.

**Figure 6:**
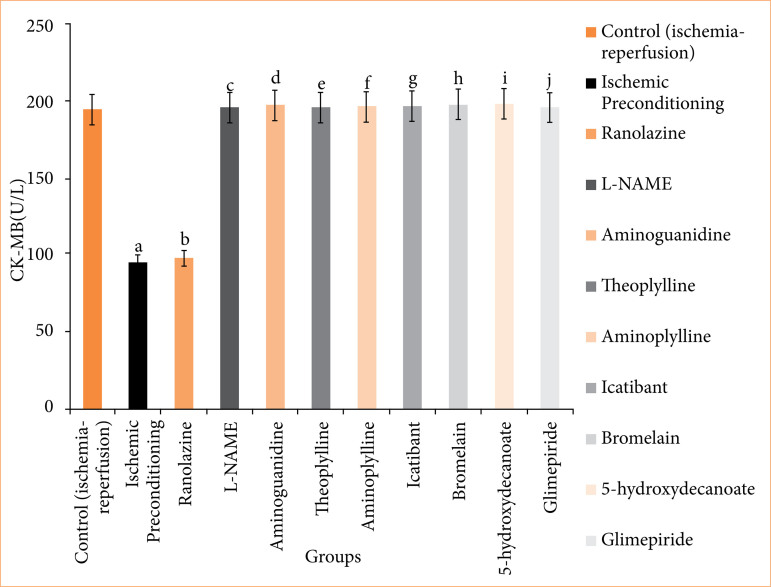
Creatine kinase myoglobin binding (CK-MB) release in coronary effluent of isolated rat heart at 120-minute reperfusion. The values presented are the mean ± standard error of the mean of 11 experimental groups, with six in each experimental group. Data were statistically analyzed by using one-way analysis of variance followed by Tukey’s multiple comparisons range test. In the context of significance, p > 0.05 indicates a non-significant value, *p ≤ 0.05 denotes a significant value, **p < 0.01 denotes a very significant value, and ***p < 0.001 denotes an extremely significant value. Notations such as c, d, e, f, g, h, i, and j indicate comparisons between ranolazine alone group (p < 0.05), in which a comparison marked with a and b letter denotes statistical significance versus control (ischemia-reperfusion) (p < 0.05). All combined treatments show significant differences from ranolazine alone (p < 0.05) and are not different from the control (p > 0.05).

Source: Elaborated by the authors.

**Figure 7:**
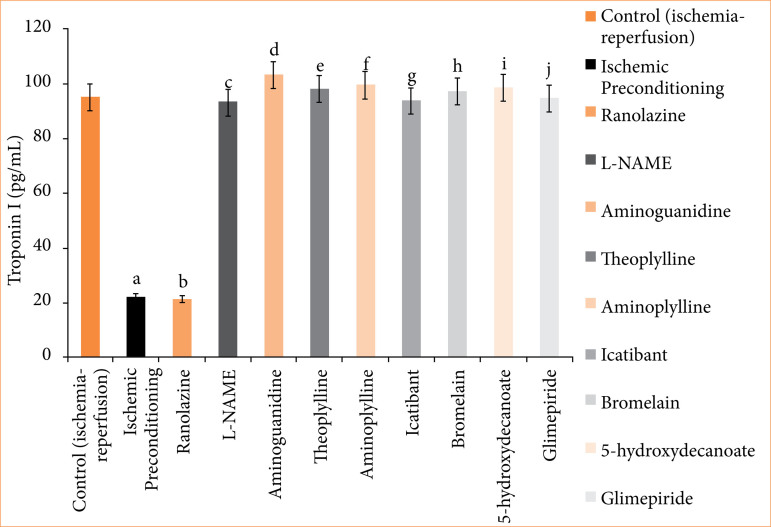
Troponin-I (pg/mL) release in coronary effluent of isolated rat heart at 120-minute reperfusion. The values presented are the mean ± standard error of the mean of 11 experimental groups, with six in each experimental group. Data were statistically analyzed by using one-way analysis of variance followed by Tukey’s multiple comparisons range test. In the context of significance, p > 0.05 indicates a non-significant value, *p ≤ 0.05 denotes a significant value, **p < 0.01 denotes a very significant value, and ***p < 0.001 denotes an extremely significant value. Notations such as c, d, e, f, g, h, i, and j indicate comparisons between ranolazine alone group (p < 0.05), in which a comparison marked with a and b letter denotes statistical significance versus control (ischemia-reperfusion) (p < 0.05). All combined treatments show significant differences from ranolazine alone (p < 0.05) and are not different from the control (p > 0.05).

Source: Elaborated by the authors.

**Figure 8:**
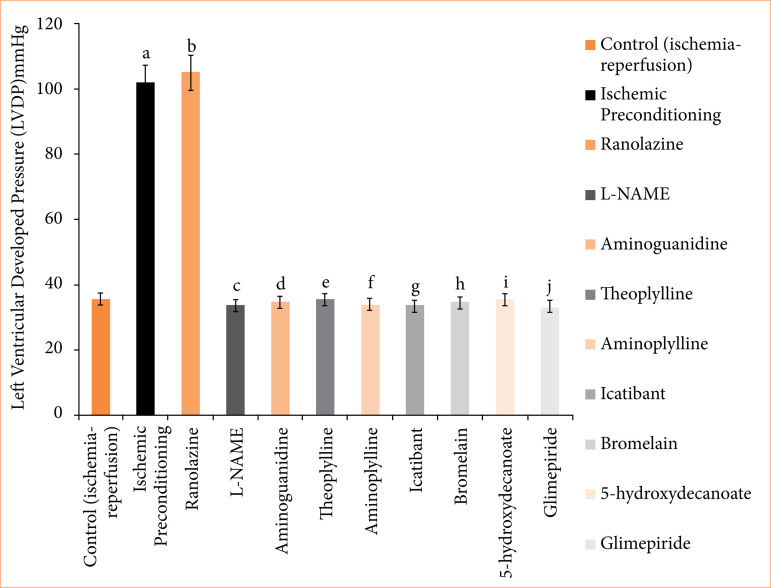
Left ventricular developed pressure (mmHg) of isolated rat heart at 120-minute reperfusion. The values presented are the mean ± standard error of the mean of 11 experimental groups, with six in each experimental group. Data were statistically analyzed by using one-way analysis of variance followed by Tukey’s multiple comparisons range test. In the context of significance, p > 0.05 indicates a non-significant value, *p ≤ 0.05 denotes a significant value, **p < 0.01 denotes a very significant value, and ***p < 0.001 denotes an extremely significant value. Notations such as c, d, e, f, g, h, i, and j indicate comparisons between ranolazine alone group (p < 0.05), in which a comparison marked with a and b letter denotes statistical significance versus control (ischemia-reperfusion) (p < 0.05). All combined treatments show significant differences from ranolazine alone (p < 0.05) and are not different from the control (p > 0.05).

Source: Elaborated by the authors.

**Figure 9:**
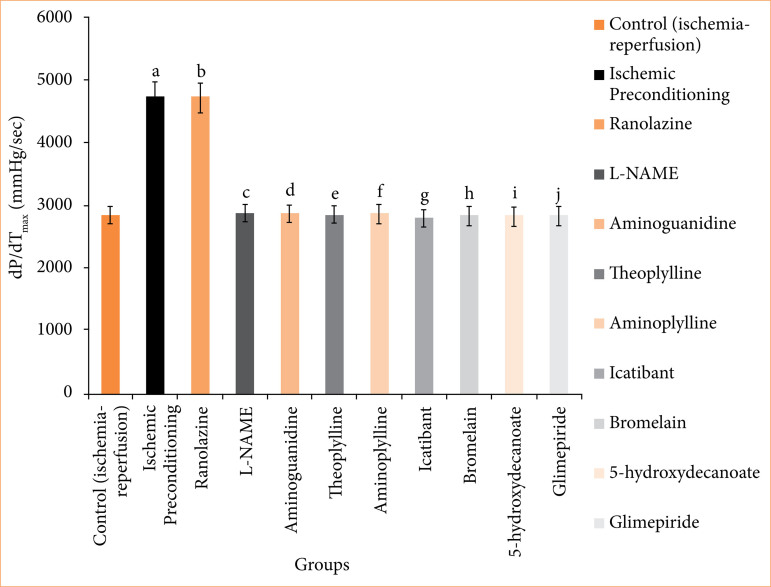
Left ventricular dP/dT_max_ (mmHg/sec) [maximum rate of contraction] of isolated rat heart at 120-minute reperfusion. The values presented are the mean ± standard error of the mean of 11 experimental groups, with six in each experimental group. Data were statistically analyzed by using one-way analysis of variance followed by Tukey’s multiple comparisons range test. In the context of significance, *p* > 0.05 indicates a non-significant value, *p ≤ 0.05 denotes a significant value, **p < 0.01 denotes a very significant value, and ***p < 0.001 denotes an extremely significant value. Notations such as c, d, e, f, g, h, i, and j indicate comparisons between ranolazine alone group (*p* < 0.05), in which a comparison marked with a and b letter denotes statistical significance versus control (ischemia-reperfusion) (*p* < 0.05). All combined treatments show significant differences from ranolazine alone (*p* < 0.05) and are not different from the control (*p* > 0.05).

Source: Elaborated by the authors.

**Figure 10:**
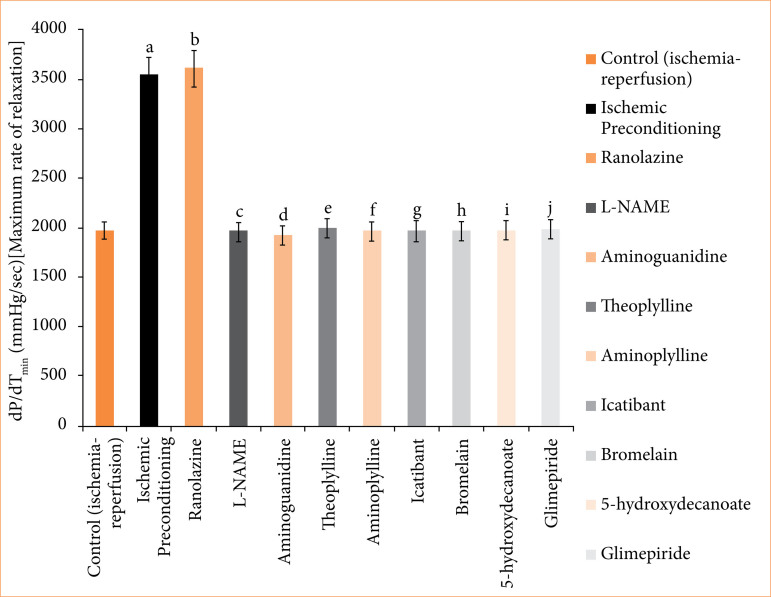
Left ventricular dP/dT_min_ (mmHg/sec) [maximum rate of relaxation] of isolated rats’ heart at 120-minute reperfusion. The values presented are the mean ± standard error of the mean of 11 experimental groups, with six in each experimental group. Data were statistically analyzed by using one-way analysis of variance followed by Tukey’s multiple comparisons range test. In the context of significance, *p* > 0.05 indicates a non-significant value, *p ≤ 0.05 denotes a significant value, **p < 0.01 denotes a very significant value, and ***p < 0.001 denotes an extremely significant value. Notations such as c, d, e, f, g, h, i, and j indicate comparisons between ranolazine alone group (*p* < 0.05), in which a comparison marked with a and b letter denotes statistical significance versus control (ischemia-reperfusion) (*p* < 0.05). All combined treatments show significant differences from ranolazine alone (*p* < 0.05) and are not different from the control (*p* > 0.05).

Source: Elaborated by the authors.

In the ischemic preconditioning group, there was a significant decrease in myocardial infarct size compared to the control (ischemia-reperfusion) group. The myocardial infarction in the control group was measured at 65.667 ± 0.558%, whereas in the ischemic preconditioning group, it was 26.167 ± 0.872% (p < 0.05). LDH levels in the control group were recorded at 155.500 ± 0.958 I.U., while in the ischemic preconditioning group, LDH levels were notably lower at 101.667 ± 2.789 I.U. (*p* < 0.05). Similarly, CK-MB levels in the control group were 198.500 ± 1.803 I.U., whereas in the ischemic preconditioning group, they were significantly reduced to 97.167 ± 1.721 I.U. (*p* < 0.05). Troponin I levels in the control group were recorded at 95.333 ± 7.688, while in the ischemic preconditioning group, troponin I levels were notably lower at 22.33333 ± 3.721 pg/mL (*p* < 0.05). In control (ischemia-reperfusion) group, cardiac mechanical parameters were: LVDP: 35.667 ± 1.726; dP/dT_max_: 2855.833 ± 117.385 mmHg/sec; and dP/dT_min_: 1968.167 ± 71.657 mmHg/sec. They were significantly different from the parameters of the ischemic preconditioning group: LVDP: 102.167 ± 3.177 mmHg; dP/dT_max_: 4743.333 ± 107.317 mmHg/sec; dP/dT_min_: 3,546.333 ± 32.444 mmHg/sec (*p* < 0.05) (Figs. 4-10, Tables 3-9).

**Table 3:** Myocardial infarct size of different group of animals^<tfn href="tfn02">#</tfn>^.

Groups	Groups description	Infarct size
I	Control (ischemia-reperfusion)	65.667 ± 0.558
II	Ischemic preconditioning	26.167 ± 0.872***a
III	Ranolazine	26.833 ± 1.046***b
IV	Ranolazine + L-NAME	62.333 ± 1.022***c
V	Ranolazine + aminoguanidine	63.667 ± 0.988***d
VI	Ranolazine + theoplylline	63.500 ± 1.565***e
VII	Ranolazine + aminoplylline	63.833 ± 0.946***f
VIII	Ranolazine + icatibant	63.667 ± 1.453***g
IX	Ranolazine + bromelain	63.333 ± 0.803***h
X	Ranolazine + 5-hydroxydecanoate	63.167 ± 1.046***i
XI	Ranolazine + glimepiride	62.833 ± 1.014***j

# The values presented are the mean ± standard error of the mean of 11 experimental groups, with six in each experimental group.

Data were statistically analyzed by using one-way analysis of variance followed by Tukey’s multiple comparisons range test. In the context of significance, *p* > 0.05 indicates a non-significant value, *p ≤ 0.05 denotes a significant value, **p < 0.01 denotes a very significant value, and

*** *p* < 0.001 denotes an extremely significant value. Notations such as c, d, e, f, g, h, i, and j indicate comparisons between ranolazine alone group (*p* < 0.05), where a comparison marked with a and b letter denotes statistical significance verses control (ischemia-reperfusion) (*p* < 0.05). All combined treatments show significant differences from ranolazine alone (*p* < 0.05) and are not different from the control (*p* > 0.05).

Source: Elaborated by the authors.

**Table 4:** Lactate dehydrogenase (LDH) release in coronary effluent of isolated rat heart at 120-minute reperfusion^<tfn href="tfn04">#</tfn>^.

Groups	Groups description	Lactate dehydrogenase (LDH) at 120-minute reperfusion
I	Control (ischemia-reperfusion)	155.667 ± 0.988
II	Ischemic preconditioning	101.667 ± 2.789***a
III	Ranolazine	101.500 ± 1.147***b
IV	Ranolazine + L-NAME	154.667 ± 1.256***c
V	Ranolazine + aminoguanidine	154.833 ± 1.138***d
VI	Ranolazine + theoplylline	155.333 ± 1.856***e
VII	Ranolazine + aminoplylline	154.833 ± 1.138***)f
VII	Ranolazine + icatibant	154.167 ± 1.195***g
IX	Ranolazine + bromelain	155.167 ± 1.195***h
X	Ranolazine + 5-hydroxydecanoate	154.667 ± 1.054***i
XI	Ranolazine + glimepiride	155.833 ± 1.352***j

# The values presented are the mean ± standard error of the mean of 11 experimental groups, with six in each experimental group.

Data were statistically analyzed by using one-way analysis of variance followed by Tukey’s multiple comparisons range test. In the context of significance, *p* > 0.05 indicates a non-significant value, *p ≤ 0.05 denotes a significant value, **p < 0.01 denotes a very significant value, and

*** *p* < 0.001 denotes an extremely significant value. Notations such as c, d, e, f, g, h, i, and j indicate comparisons between ranolazine alone group (*p* < 0.05), in which a comparison marked with a and b letter denotes statistical significance verses control (ischemia-reperfusion) (*p* < 0.05). All combined treatments show significant differences from ranolazine alone (*p* < 0.05) and are not different from the control (*p* > 0.05).

Source: Elaborated by the authors.

**Table 5:** Creatine kinase myoglobin binding (CK-MB) release in coronary effluent of isolated rat heart at 120-minute reperfusion^<tfn href="tfn06">#</tfn>^.

Groups	Groups description	Lactate dehydrogenase (LDH) at 120-minute reperfusion
I	Control (ischemia-reperfusion)	198.500 ± 1.803
II	Ischemic preconditioning	97.167 ± 1.721***a
III	Ranolazine	100.167 ± 1.302***b
IV	Ranolazine + L-NAME	200.167 ± 1.537***c
V	Ranolazine + aminoguanidine	198.333 ± 1.145***d
VI	Ranolazine + theoplylline	199.833 ± 1.621***e
VII	Ranolazine + aminoplylline	200.167 ± 1.833***f
VII	Ranolazine + icatibant	200.500 ± 1.607***g
IX	Ranolazine + bromelain	201.333 ± 1.563***h
X	Ranolazine + 5-hydroxydecanoate	201.833 ± 1.815***i
XI	Ranolazine + glimepiride	199.833 ± 1.579***j

# The values presented are the mean ± standard error of the mean of 11 experimental groups, with six in each experimental group.

Data were statistically analyzed by using one-way analysis of variance followed by Tukey’s multiple comparisons range test. In the context of significance, *p* > 0.05 indicates a non-significant value, *p ≤ 0.05 denotes a significant value, **p < 0.01 denotes a very significant value, and

*** ***p < 0.001 denotes an extremely significant value. Notations such as c, d, e, f, g, h, i, and j indicate comparisons between ranolazine alone group (*p* < 0.05), in which a comparison marked with a and b letter denotes statistical significance *versus* control (ischemia-reperfusion) (*p* < 0.05). All combined treatments show significant differences from tanolazine alone (*p* < 0.05) and are not different from the control (*p* > 0.05).

Source: Elaborated by the authors.

**Table 6:** Troponin-I (pg/mL) of different group of animals’ release in coronary effluent of isolated rat heart at 120-minute reperfusion^<tfn href="tfn08">#</tfn>^.

Groups	Groups description	Troponin-I (pg/mL)
I	Control (ischemia-reperfusion)	95.333 ± 7.688
II	Ischemic preconditioning	22.33333 ± 3.721***a
III	Ranolazine	21.667 ± 2.155***b
IV	Ranolazine + L-NAME	93.333 ± 8.028***c
V	Ranolazine + aminoguanidine	103.333 ± 8.028***d
VI	Ranolazine + theoplylline	98.167 ± 7.391***e
VII	Ranolazine + aminoplylline	99.667 ± 6.349***f
VIII	Ranolazine + icatibant	93.833 ± 7.875***g
IX	Ranolazine + bromelain	97.333 ± 5.678***h
X	Ranolazine + 5-hydroxydecanoate	98.667 ± 8.586***i
XI	Ranolazine + glimepiride	99.667 ± 6.349***j

# The values presented are the mean ± standard error of the mean of 11 experimental groups, with six in each experimental group.

Data were statistically analyzed by using one-way analysis of variance followed by Tukey’s multiple comparisons range test. In the context of significance, *p* > 0.05 indicates a non-significant value, *p ≤ 0.05 denotes a significant value, **p < 0.01 denotes a very significant value, and

*** *p* < 0.001 denotes an extremely significant value. Notations such as c, d, e, f, g, h, i, and j indicate comparisons between ranolazine alone group (*p* < 0.05), in which a comparison marked with a and b letter denotes statistical significance versus control (ischemia-reperfusion) (*p* < 0.05). All combined treatments show significant differences from ranolazine alone (*p* < 0.05) and are not different from the control (*p* > 0.05).

Source: Elaborated by the authors.

**Table 7:** Left ventricular developed pressure (mmHg) of different group of animals^<tfn>#</tfn>^.

Groups	Groups description	Left ventricular developed pressure (mmHg)
I	Control (ischemia-reperfusion)	35.667 ± 1.726
II	Ischemic preconditioning	102.167 ± 3.177***a
III	Ranolazine	105.167 ± 4.285***b
IV	Ranolazine + L-NAME	33.667 ± 1.429***c
V	Ranolazine + aminoguanidine	34.667 ± 2.591***d
VI	Ranolazine + theoplylline	35.167 ± 2.197***e
VII	Ranolazine + aminoplylline	34.167 ± 2.959***f
VIII	Ranolazine + icatibant	33.667 ± 1.429***g
IX	Ranolazine + bromelain	34.667 ± 2.59058***h
X	Ranolazine + 5-hydroxydecanoate	35.333 ± 2.347576***i
XI	Ranolazine + glimepiride	33.333 ± 1.874***j

# The values presented are the mean ± standard error of the mean of 11 experimental groups, with six in each experimental group.

Data were statistically analyzed by using one-way analysis of variance followed by Tukey’s multiple comparisons range test. In the context of significance, *p* > 0.05 indicates a non-significant value, *p ≤ 0.05 denotes a significant value, **p < 0.01 denotes a very significant value, and

*** *p* < 0.001 denotes an extremely significant value. Notations such as c, d, e, f, g, h, i, and j indicate comparisons between ranolazine alone group (*p* < 0.05), in which a comparison marked with a and b letter denotes statistical significance versus control (ischemia-reperfusion) (*p* < 0.05). All combined treatments show significant differences from ranolazine alone (*p* < 0.05) and are not different from the control (*p* > 0.05).

Source: Elaborated by the authors.

**Table 8:** Left ventricular dP/dT_max_ (mmHg/sec) [maximum rate of contraction] of different group of animals^<tfn href="tfn12">#</tfn>^.

Groups	Groups description	Left Ventricular dP/dTmax (mmHg/sec) [maximum rate of contraction]
I	Control (ischemia-reperfusion)	2,855.833 ± 117.385
II	Ischemic preconditioning	4,743.333 ± 107.317***a
III	Ranolazine	4,729.5 ± 74.031***b
IV	Ranolazine + L-NAME	2,980 ± 74.935***c
V	Ranolazine + aminoguanidine	2,945 ± 59.663***d
VI	Ranolazine + theoplylline	2,861.333 ± 73.741***e
VII	Ranolazine + aminoplylline	2,880.833 ± 60.619***f
VIII	Ranolazine + icatibant	2,807.833 ± 75.094***g
IX	Ranolazine + bromelain	2,844.667 ± 60.456***h
X	Ranolazine + 5-hydroxydecanoate	2,905.833 ± 89.328***i
XI	Ranolazine + glimepiride	2,964.833 ± 50.275***j

# The values presented are the mean ± standard error of the mean of 11 experimental groups, with six in each experimental group.

Data were statistically analyzed by using one-way analysis of variance followed by Tukey’s multiple comparisons range test. In the context of significance, *p* > 0.05 indicates a non-significant value, *p ≤ 0.05 denotes a significant value, **p < 0.01 denotes a very significant value, and

*** *p* < 0.001 denotes an extremely significant value. Notations such as c, d, e, f, g, h, i, and j indicate comparisons between ranolazine alone group (*p* < 0.05), in which a comparison marked with a and b letter denotes statistical significance versus control (ischemia-reperfusion) (*p* < 0.05). All combined treatments show significant differences from ranolazine alone (*p* < 0.05) and are not different from the control (*p* > 0.05).

Source: Elaborated by the authors.

**Table 9:** Left ventricular dP/dT_min_ (mmHg/sec) [maximum rate of relaxation] of isolated rats’ heart of different group of animals^#^.

Groups	Groups description	Left ventricular dP/dTmax (mmHg/sec) [maximum rate of contraction]
I	Control (ischemia-reperfusion)	1,968.167 ± 71.657
II	Ischemic preconditioning	3,546.333 ± 32.444***a
III	Ranolazine	3,612.667 ± 67.757***b
IV	Ranolazine + L-NAME	2,040.333 ± 77.276***c
V	Ranolazine + aminoguanidine	2,055.667 ± 76.766***d
VI	Ranolazine + theoplylline	1,991.5 ± 70.975***e
VII	Ranolazine + aminoplylline	1,964.833 ± 81.159***f
VIII	Ranolazine + icatibant	1,978.167 ± 76.048***g
IX	Ranolazine + bromelain	1,964.5 ± 46.353***h
X	Ranolazine + 5-hydroxydecanoate	2,019.333 ± 67.64351***i
XI	Ranolazine + glimepiride	2,099.667 ± 86.979***j

# The values presented are the mean ± standard error of the mean of 11 experimental groups, with six in each experimental group.

Data were statistically analyzed by using one-way analysis of variance followed by Tukey’s multiple comparisons range test. In the context of significance, *p* > 0.05 indicates a non-significant value, *p ≤ 0.05 denotes a significant value, **p < 0.01 denotes a very significant value, and

*** *p* < 0.001 denotes an extremely significant value. Notations such as c, d, e, f, g, h, i, and j indicate comparisons between ranolazine alone group (*p* < 0.05), in which a comparison marked with a and b letter denotes statistical significance versus control (ischemia-reperfusion) (*p* < 0.05). All combined treatments show significant differences from ranolazine alone (*p* < 0.05) and are not different from the control (*p* > 0.05).

Source: Elaborated by the authors.

### Effect of control (ischemia-reperfusion) and ranolazine treatment group on infarct size, LDH, CK-MB, troponin I, LVDP, dP/dT_max_, and dP/dT_min_

In the ranolazine treatment group, there was a significant reduction in myocardial infarct size compared to the control (ischemia-reperfusion) group. The myocardial infarction in the control group measured 65.667 ± 0.558%, whereas in the ranolazine treatment group, it was notably lower at 5.334 ± 0.422% (*p* < 0.05). LDH levels in the control group were recorded at 155.500 ± 0.958 I.U., whereas in the ranolazine treatment group, LDH levels were significantly decreased to 101.500 ± 1.147 I.U. (*p* < 0.05). Similarly, CK-MB levels in the control group were 198.500 ± 1.803 I.U., whereas in the ranolazine treatment group, they were notably reduced to 100.167 ± 1.302 I.U. (p < 0.05). Troponin I levels in the control group were recorded at 95.333 ± 7.688 pg/mL, whereas in the ranolazine treatment group, troponin I levels were significantly decreased to 21.667 ± 2.155 pg/mL (*p* < 0.05). In control (ischemia-reperfusion) group, cardiac mechanical parameters were: LVDP: 35.667 ± 1.726; dP/dT_max_: 2,855.833 ± 117.385 mmHg/sec; and dP/dT_min_: 1,968.167 ± 71.657 mmHg/sec, which has significant difference from ranolazine treatment group: LVDP: 105.167 ± 4.285 mmHg; dP/dT_max_: 4,729.5 ± 74.031 mmHg/sec; dP/dT_min_: 3,612.667 ± 67.757 mmHg/sec for ranolazine (*p* < 0.05) (Figs. 4-10, Tables 3-9).

### Effect of ranolazine and ranolazine + L-NAME (nitric oxide synthase inhibitor) on infarct size, LDH, CK-MB, troponin I, LVDP, dP/dT_max_, and dP/dT_min_

Combining L-NAME, a nitric oxide synthase inhibitor, with ranolazine results in an increase in myocardial infarct size compared to treatment with ivabradine alone. The myocardial infarction size in the ranolazine + L-NAME group was measured at 62.333 ± 1.022%, while in the ranolazine treatment group, it was significantly lower, at 26.833 ± 1.046% (*p* < 0.05). LDH levels in the ranolazine + L-NAME group were recorded at 154.667 ± 1.256 I.U., whereas in the ranolazine treatment group, LDH levels were significantly lower, at 101.500 ± 1.147 I.U. (*p* < 0.05). Similarly, CK-MB levels in the ranolazine + L-NAME group were elevated at 200.167 ± 1.537 I.U., whereas in the ranolazine treatment group, they were notably lower at 100.167 ± 1.302 I.U. (*p* < 0.05). Troponin I levels in the ranolazine + L-NAME group were elevated at 93.333 ± 8.028 pg/mL, whereas in the ranolazine treatment group, they were notably lower at 21.667 ± 2.155 pg/mL (*p* < 0.05). In ranolazine + L-NAME group, the parameters were: LVDP: 33.667 ± 1.429mmHg; dP/dT_max_: 2,980 ± 74.935 mmHg/sec; and dP/dT_min_: 2,040.333 ± 77.276 mmHg/sec, which has significant difference from ranolazine treatment group: LVDP: 105.167 ± 4.285 mmHg; dP/dT_max_: 4,729.5 ± 74.031 mmHg/sec; and dP/dT_min_: 3,612.667 ± 67.757 mmHg/sec for ranolazine (p < 0.05) (Figs. 4-10, Tables 3-9).

### Effect of ranolazine and ranolazine + aminoguanidine (nitric oxide synthase inhibitor) on infarct size, LDH, CK-MB, troponin I, LVDP, dP/dT_max_, and dP/dT_min_

Combining aminoguanidine, an inducible nitric oxide synthase (iNOS) inhibitor, with ranolazine leads to an increase in myocardial infarct size compared to treatment with ranolazine alone. The myocardial infarction size in the ranolazine + aminoguanidine group was measured at 63.667 ± 0.988%, while in the ranolazine treatment group, it was significantly lower at 26.833 ± 1.046% (*p* < 0.05). LDH levels in the ranolazine + aminoguanidine group were recorded at 154.833 ± 1.138 I.U., whereas in the ivabradine treatment group, LDH levels were significantly lower at 101.500 ± 1.147 I.U. (*p* < 0.05). Similarly, CK-MB levels in the ranolazine + aminoguanidine group were elevated at 198.333 ± 1.145 I.U., whereas in the ranolazine treatment group, they were notably lower at 100.167 ± 1.302 I.U. (*p* < 0.05). Troponin I levels in the ranolazine + aminoguanidine group were elevated at 103.333 ± 8.028 pg/mL, whereas in the ivabradine treatment group, they were notably lower at 21.667 ± 2.155 pg/mL (*p* < 0.05). In ranolazine + aminoguanidine group, the parameters were: LVDP: 34.667 ± 2.591 mmHg; dP/dT_max_: 2,945 ± 59.663 mmHg/sec; and dP/dT_min_: 2,055.667 ± 76.766 mmHg/sec, which has significant difference from ranolazine treatment group, whose parameters were: LVDP: 105.167 ± 4.285 mmHg; dP/dT_max_: 4,729.5 ± 74.031 mmHg/sec; and dP/dT_min_: 3,612.667 ± 67.757 mmHg/sec for ranolazine (*p* < 0.05) (Figs. 4-10, Tables 3-9).

### Effect of ranolazine and ranolazine + theophylline (adenosine inhibitor) on infarct size, LDH, CK-MB, troponin I, LVDP, dP/dT_max_, and dP/dT_min_

Combining theophylline, an adenosine inhibitor, with ranolazine results in extremely significant values in myocardial infarct size compared to treatment with ranolazine alone. The myocardial infarction size in the ranolazine + theophylline group was measured at 63.500 ± 1.565%, while in the ranolazine treatment group, it was at 26.833 ± 1.046% (*p* < 0.05). LDH levels in the ranolazine + theophylline group were recorded at 155.333 ± 1.856I.U., whereas in the ranolazine treatment group, LDH levels were 101.500 ± 1.147 I.U. (*p* < 0.05). Similarly, CK-MB levels in the ranolazine + theophylline group was 199.833 ± 1.621 I.U., whereas in the ranolazine treatment group, was at 100.167 ± 1.302 I.U. (*p* < 0.05). Troponin I levels in the ranolazine + theophylline group was 98.167 ± 7.391 pg/mL, whereas in the ranolazine treatment group, they were notably lower at 21.667 ± 2.155 pg/mL (*p* < 0.05). In ranolazine + theophylline group, mechanical parameters of rat heart were: LVDP: 35.167 ± 2.197 mmHg; dP/dT_max_: 2,861.333 ± 73.741 mmHg/sec; and dP/dT_min_: 1,991.5 ± 70.975 mmHg/sec, which has significant difference from ranolazine treatment group, whose parameters were: LVDP: 105.167 ± 4.285 mmHg; dP/dT_max_: 4,729.5 ± 74.031 mmHg/sec; and dP/dT_min_: 3,612.667 ± 67.757 mmHg/sec for ranolazine (*p* < 0.05) (Figs. 4-10, Tables 3-9).

### Effect of ranolazine and ranolazine + aminophylline (adenosine inhibitor) on infarct size, LDH, CK-MB, troponin I, LVDP, dP/dT_max_, and dP/dT_min_

Combining aminophylline, an adenosine inhibitor, with ranolazine leads extremely significant values in myocardial infarct size compared to treatment with ranolazine alone. The myocardial infarction size in the ranolazine + aminophylline group was measured at 63.833 ± 0.946%, while in the ranolazine treatment group, it was 26.833 ± 1.046% (*p* < 0.05). LDH levels in the ranolazine + aminophylline group were recorded at 154.833 ± 1.138 I.U., whereas in the ranolazine treatment group, LDH levels was 101.500 ± 1.147 I.U. (*p* < 0.05). Similarly, CK-MB levels in the ranolazine + aminophylline group were elevated at 200.167 ± 1.833 I.U., whereas in the ranolazine treatment group, they were 100.167 ± 1.302 I.U. (*p* < 0.05). Troponin I levels in the ranolazine + aminophylline group was 99.667 ± 6.349 pg/mL, whereas in the ranolazine treatment group, it was 21.667 ± 2.155 pg/mL (*p* < 0.05). In ranolazine + aminophylline group, mechanical parameters of rat heart were: LVDP: 34.167 ± 2.959 mmHg; dP/dT_max_: 2,880.833 ± 60.619 mmHg/sec; and dP/dT_min_: 1,964.833 ± 81.159 mmHg/sec, which has significant difference from ranolazine treatment group parameters: LVDP: 105.167 ± 4.285 mmHg; dP/dT_max_: 4,729.5 ± 74.031 mmHg/sec; and dP/dT_min_: 3,612.667 ± 67.757 mmHg/sec (*p* < 0.05) (Figs. 4-10, Tables 3-9).

### Effect of ranolazine and ranolazine + icatibant (bradykinin B2 antagonist) on infarct size, LDH, CK-MB, troponin I, LVDP, dP/dT_max_, and dP/dT_min_

When icatibant (bradykinin B2 antagonist) is combined with ranolazine, there was extremely significant increase in myocardial infarct size compared to treatment with ranolazine alone. The myocardial infarction size in the ranolazine + icatibant group was measured at 63.667 ± 1.4%, while in the ranolazine treatment group, it was comparable at 26.833 ± 1.046% (*p* < 0.05). LDH levels in the ranolazine + icatibant group were recorded at 154.167 ± 1.195 I.U., whereas in the ranolazine treatment group, LDH levels was 101.500 ± 1.147 I.U. (*p* < 0.05). Similarly, CK-MB levels in the ranolazine + icatibant group were 200.500 ± 1.6079 I.U., whereas in the ranolazine treatment group, they were also similar at 100.167 ± 1.302 I.U. (*p* < 0.05). Troponin I levels in the ranolazine + icatibant group was 93.833 ± 7.875 pg/mL, whereas in the ranolazine treatment group, it was 21.667 ± 2.155 pg/mL (*p* < 0.05). In ranolazine + icatibant group, mechanical parameters of rat heart were: LVDP: 33.667 ± 1.429 mmHg; dP/dT_max_: 2,807.833 ± 75.094 mmHg/sec; and dP/dT_min_: 1,978.167 ± 76.048 mmHg/sec, which has significant difference from ranolazine treatment group parameters: LVDP: 105.167 ± 4.285 mmHg; dP/dT_max_: 4,729.5 ± 7 4.031 mmHg/sec; and dP/dT_min_: 3,612.667 ± 67.757 mmHg/sec for ranolazine (*p* < 0.05) (Figs. 4-10, Tables 3-9).

### Effect of ranolazine and ranolazine + bromelain (kinins-bradykinin inhibitor) on infarct size, LDH, CK-MB, troponin I, LVDP, dP/dT_max_, and dP/dT_min n_

When bromelain, a kinins-bradykinin inhibitor, is combined with ranolazine, there was significant difference in myocardial infarct size compared to treatment with ranolazine alone. The myocardial infarction size in the ranolazine + bromelain group was measured at 63.333 ± 0.803%, while in the ranolazine treatment group, it was comparable at 26.833 ± 1.046% (*p* < 0.05). LDH levels in the ranolazine + bromelain group was recorded at 155.167 ± 1.195 I.U., whereas in the ranolazine treatment group, LDH levels were 101.500 ± 1.147 I.U. *p* < 0.05). Similarly, CK-MB levels in the ranolazine + bromelain group were 201.333 ± 1.563 I.U., whereas in the ranolazine treatment group, they were also similar at 100.167 ± 1.302 I.U. (*p* < 0.05). Troponin I levels in the ranolazine + bromelain group were 97.333 ± 5.678 pg/mL, whereas in the ranolazine treatment group, they were 21.667 ± 2.155 pg/mL (*p* < 0.05). In ranolazine + bromelain group, mechanical parameters of rat heart were: LVDP: 34.667 ± 2.59 mmHg; dP/dT_max_: 2,844.667 ± 60.456 mmHg/sec; and dP/dT_min_: 1,964.5 ± 46.353 mmHg/sec, which has significant difference from ranolazine treatment group parameters: LVDP: 105.167 ± 4.285 mmHg; dP/dT_max_: 4,729.5 ± 74.031 mmHg/sec; and dP/dT_min_: 3,612.667 ± 67.757 mmHg/sec for ranolazine (*p* < 0.05) (Figs. 4-10, Tables 3-9).

### Effect of ranolazine and ranolazine + 5-hydroxydecanoate (K + ATPase inhibitor) on infarct size, LDH, CK-MB, troponin I, LVDP, dP/dT_max_, and dP/dT_min_

When 5-hydroxydecanoate, an ATP-dependent K + channel inhibitor, is combined with ranolazine, there is a significant increase in myocardial infarct size compared to treatment with ranolazine alone. The myocardial infarction size in the ranolazine + 5-hydroxydecanoate group was measured at 63.167 ± 1.046%, while in the ranolazine treatment group, it was significantly lower at 26.833 ± 1.046% (*p* < 0.05). LDH levels in the ranolazine + 5-hydroxydecanoate group were recorded at 154.667 ± 1.054 I.U., whereas in the ranolazine treatment group, LDH levels were significantly lower at 101.500 ± 1.147 I.U. (*p* < 0.05). Similarly, CK-MB levels in the ranolazine + 5-hydroxydecanoate group were elevated at 201.833 ± 1.815 I.U., whereas in the ranolazine treatment group, they were notably lower at 100.167 ± 1.302 I.U. (*p* < 0.05). Troponin I levels in the ranolazine + 5-hydroxydecanoate group were 98.667 ± 8.586 pg/mL, whereas in the ranolazine treatment group, they were 21.667 ± 2.155 pg/mL (*p* < 0.05). In ranolazine + 5-hydroxydecanoate group, mechanical parameters of rat heart were: LVDP: 35.333 ± 2.348 mmHg; dP/dT_max_: 2,905.833 ± 89.328 mmHg/sec; and dP/dT_min_: 2,019.333 ± 67.644 mmHg/sec, which has significant difference from ranolazine treatment group parameters: LVDP: 105.167 ± 4.285 mmHg; dP/dT_max_: 4,729.5 ± 74.031 mmHg/sec; and dP/dT_min_: 3,612.667 ± 67.757 mmHg/sec for ranolazine (*p* < 0.05) (Figs. 4-10, Tables 3-9).

### Effect of ranolazine and ranolazine + glimepiride (K + ATPase inhibitor) on infarct size, LDH, CK-MB, troponin I, LVDP, dP/dT_max_, and dP/dT_min_

When glimepiride, an ATP-dependent K + channel inhibitor, is combined with ranolazine, there is a significant increase in myocardial infarct size compared to treatment with ranolazine alone. The myocardial infarction size in the ranolazine + glimepiride group was measured at 62.833 ± 1.014%, while in the ranolazine treatment group, it was significantly lower at 26.833 ± 1.046% (*p* < 0.05). LDH levels in the ranolazine + glimepiride group were recorded at 155.833 ± 1.352 I.U., whereas in the ranolazine treatment group, LDH levels were significantly lower at 101.500 ± 1.147 I.U. (*p* < 0.05). Similarly, CK-MB levels in the ranolazine + glimepiride group were elevated at 199.833 ± 1.579 I.U., whereas in the ranolazine treatment group, they were notably lower at 100.167 ± 1.302 I.U. (*p* < 0.05). Troponin I levels in the ranolazine + glimepiride group were 99.667 ± 6.349 pg/mL, whereas in the ranolazine treatment group, they were 21.667 ± 2.155 pg/mL (*p* < 0.05). In ranolazine + glimepiride group, mechanical parameters of rat heart were: LVDP: 33.333 ± 1.874 mmHg; dP/dT_max_: 2,964.833 ± 50.275 mmHg/sec; and dP/dT_min_: 2,099.667 ± 86.979 mmHg/sec, which has significant difference from ranolazine treatment group parameters: LVDP: 105.167 ± 4.285 mmHg; dP/dT_max_: 4,729.5 ± 74.031 mmHg/sec; and dP/dT_min_: 3,612.667 ± 67.757 mmHg/sec for ranolazine (*p* < 0.05) (Figs. 4-10, Tables 3-9).

## Discussion

### Mechanism of action of ranolazine

Numerous studies demonstrated ranolazine as cardioproective in clinical and in animal studies. In its ranolazine ischemia Na + overload mechanical dysfunction ↑ diastolic tension ↓ contractility Ca2 + overload electrical dysfunction arrhythmias NCX ↑ late INa Na/K-ATPase NHE O2 Supply & Demand ↑ ATP consumption ↓ ATP formation. Late INa under normal and increased late INa under pathophysiological conditions, late INa inhibition with ranolazine reverse mode (usually during the action potential), it brings calcium into the cell in exchange to trans-sarcolemma elimination of sodium. The functionality and direction of movement are influenced by the protein’s quantity, along with factors such as membrane potential, intracellular sodium levels, and intracellular calcium concentrations. Increased sodium levels resulting from myocardial hypoxia (via late INa) stimulate reverse mode sodium-calcium exchange, consequently diminishing the cell’s ability to remove calcium from the cytosol^14,15^.

Ranolazine demonstrates pharmacological preconditioning and postconditioning effects in anesthetized rabbits by activating the RISK pathway. Research has shown that diabetic (db/db) mice exhibit heightened vulnerability to myocardial IRI and ventricular tachyarrhythmias. The study aimed to explore the antiarrhythmic mechanisms of ranolazine in db/db mouse hearts experiencing acute IRI. Ranolazine treatment was administered for one week before coronary artery ligation. Diabetic db/db and control db/ + mice were categorized into groups receiving either ranolazine or no treatment. The ischemia-reperfusion model involved 15-minute left coronary artery ligation followed by 10 minutes of reperfusion. *In-vivo* electrophysiological assessments revealed increased severity of ventricular tachyarrhythmias inducibility in db/db mice compared to control (db/ +) mice, which was mitigated by ranolazine. Optical mapping studies conducted in Langendorff-perfused hearts demonstrated that ranolazine notably reduced action potential duration, calcium transient duration, and calcium decay time, improved conduction homogeneity, and suppressed the induction of arrhythmogenic alternans. Western blotting analysis indicated that the expression of pThr17-phospholamban, calsequestrin 2, and voltage-gated sodium channel in the ischemia-reperfusion zone was significantly decreased in db/db mice, an effect ameliorated by ranolazine pretreatment, which may contribute to its antiarrhythmic effects in db/db mouse hearts experiencing IRI^20-22^.

### Ischemic pre-conditioning and its mediators

Ischemia refers to a condition in which there is partial or complete blockage of blood flow in a tissue or organ, leading to necrosis and cell death if left untreated. IRI occurs when blood flow is restored after a period of ischemia. Renal IRI is a major contributor to acute kidney injury and can arise in various clinical scenarios, such as surgeries involving partial nephrectomy, renal transplantation, aortic cross clamping, cardiopulmonary resuscitation, sepsis, and shock. Several interventions including hydrogen sulfide, superoxide dismutase (SOD), apocynin, allopurinol, hypothermia, ischemic preconditioning, and remote ischemic preconditioning (RIPC) have been identified to mitigate IRI. RIPC, which involves preconditioning one organ to protect another distant organ against prolonged IRI, has shown promise in reducing renal injury induced by IRI.

Brief episodes of ischemia and reperfusion lead to the generation of oxygen-derived free radicals, triggering the release of antioxidants that scavenge these free radicals. Although the precise protective mechanisms of RIPC are not fully elucidated, nitric oxide produced during RIPC is believed to play a role in protecting the heart against IRI. The cardioprotective effects of ischemic preconditioning have been linked to the involvement of various factors such as nitric oxide, adenosine, bradykinin, and ATP-dependent K + channels^20-44^.

### Previous findings about creatine phosphokinase myoglobin and lactate dehydrogenase in cardioprotection

Despite their limitations as cardiac biomarkers, CK-MB and LDH are extensively utilized to evaluate the degree of myocardial damage and IRI. In the realm of cardioprotective research, they serve as crucial indicators of the efficacy of diverse interventions, encompassing preparatory measures, pharmacological agents, and other therapeutic modalities aimed at mitigating tissue damage during ischemic events. In summary, both CK-MB and LDH play pivotal roles as indicators of myocardial and cellular damage in studies focused on cardioprotection. Monitoring their levels can provide insights into the effects of various strategies and interventions aimed at minimizing harm in ischemia-reperfusion injury^45,46^.

Ischemic preconditioning and ranolazine have exhibited cardioprotective effects by reducing LDH and CK-MB levels in cardiac injury induced by ischemia-reperfusion. Previous literature suggests that ischemic preconditioning achieves cardioprotection through pathways involving nitric oxide, adenosine, bradykinin, and ATP-dependent K + channels. Our research aimed to investigate whether ranolazine also engages these pathways to confer cardioprotection. Notably, it has been established that ranolazine-induced cardioprotection in dogs involves increased adenosine levels^47^.

Outcome measures in proof-of-concept clinical studies, perioperative release of cardiac ischemic biomarkers, such as CK, preferably as the myocardial fraction CK-MB, TnT or TnI is the only way to assess the efficacy of the novel cardioprotective strategy on myocardial injury. One must, however, be aware that cardiac surgery including myocardial incision or coronary clamping (*e.g.*, coronary bypass) will *per se* cause cardiac enzyme release, hence alter interpretation of the data. Other clinical endpoints include inotrope score, duration of mechanical ventilation, length of intensive care unit and hospital stay, left ventricular ejection fraction, acute kidney injury, cognitive function, cardiovascular mortality, and hospitalization for heart failure at 30 days and one year. Although there is no direct link between perioperative cardioprotection and the need for coronary revascularization, and heart failure is not included, a frequently used clinical endpoint is a composite endpoint of the rate of MACCE (death from cardiovascular causes, nonfatal myocardial infarction, coronary revascularization, or stroke), assessed within 12 months after randomization. An independent event validation committee must validate all primary events^48^.

Ischemia duration in rats is quite standardized, and 30 min of ischemia is the duration of choice. The duration of reperfusion in rats is also quite standardized. Reperfusion for 120 min is sufficient for experiments investigating acute effects of myocardial infarction. Systolic and diastolic left ventricular function deficits can be present as early as 3 hours after coronary occlusion, while after three or four weeks there is hypertrophy, which manifests in elevated left-ventricular end-diastolic pressure and right ventricular systolic pressure. Reperfusion for 120 min is the minimum time for infarct size determination in cardioprotection experiments. Keep in mind that shrinkage of the infarcted area with scar formation is observed in long-term experiments (months)^49^.

Infarct size is the primary endpoint of most of studies on myocardial infarction in rats. TTC staining is the widely accepted method to measure infarct size. Transthoracic echocardiography, cardiac magnetic resonance (CMR), and positron emission tomography have also been used in studies of myocardial infarction in rats^49^.

While early coronary reperfusion via primary percutaneous coronary intervention is established as the most efficacious therapy for minimizing infarct size in acute ST-elevation myocardial infarction (STEMI), the restoration of blood flow also introduces myocardial IRI, leading to cardiomyocyte death. Among diverse methods, ischemic conditioning, achieved through repetitive cycles of ischemia and reperfusion, has emerged as the most promising method to mitigate IRI. Ischemic conditioning can be performed by applying the protective stimulus directly to the affected myocardium or indirectly to non-affected tissue, which is known as remote ischemic conditioning (RIC). In clinical practice, RIC is often applied by serial inflations and deflations of a blood pressure cuff on a limb. Despite encouraging preclinical studies, as well as clinical studies demonstrating reductions in enzymatic infarct size (IS) and myocardial injury on imaging, the observed impact on clinical outcome has been disappointing so far. Nevertheless, previous studies indicated a potential benefit of ischemic conditioning in high-risk STEMI patients. Additional research is needed to evaluate the impact of ischemic conditioning in such high-risk cohorts. The objective of this review was to summarize the pathophysiological background and preclinical and clinical data of IRI reduction by ischemic conditioning^8^.

Despite impressive improvements in the care of patients with ST-segment elevation myocardial infarction, mortality remains high. Reperfusion is necessary for myocardial salvage, but the abrupt return of flow sets off a cascade of injurious processes that can lead to further necrosis. This has been termed myocardial IRI and is the subject of this review. The pathologic and molecular bases for myocardial IRI are increasingly understood and include injury from reactive oxygen species, inflammation, calcium overload, endothelial dysfunction, and impaired microvascular flow. A variety of pharmacologic strategies have been developed that have worked well in preclinical models and some have shown promise in the clinical setting. In addition, there are newer mechanical approaches including mechanical unloading of the heart prior to reperfusion that are in current clinical trials. Myocardial IRI (MIRI) exacerbates myocardial necrosis in patients with STEMI, contributing to mortality. It is mediated by varying combinations of reactive oxygen species, inflammation, endothelial dysfunction, impaired microvascular flow, and other factors. A variety of pharmacologic and mechanical strategies that mitigate MIRI in preclinical models have shown promise in clinical trials^50^.

### Down-regulation of plasma kininogen

Bromelain has been shown to down-regulate plasma kininogen levels. Kininogens are precursors to kinins, which are potent mediators of inflammation and vasodilation. By reducing plasma kininogen levels, bromelain may inhibit the production of kinins, thereby attenuating inflammation and its associated symptoms such as pain and swelling^51^.

Angiotensin converting-enzyme (ACE) inhibitors attenuate cardiac hypertrophy and prolong survival in animal models and patients after myocardial infarction. Considering the dual function of the ACE, the therapeutic efficacy of ACE inhibitors after myocardial infarction implicates the renin-angiotensin system and/or the kallikrein-kinin system in the pathophysiology of post-infarction cardiac remodeling. We evaluated the role of kinins, and their potential contribution to the antiremodelling effects of ACE inhibition in this setting. Rats underwent coronary artery ligation followed by chronic B2 kinin receptor blockade with icatibant (HOE 140). Additional groups of myocardial infarction rats were treated with the ACE inhibitor lisinopril, alone or in combination with icatibant. B2 kinin receptor blockade enhanced the deposition of collagen (morphometric analysis) in the left ventricular interstitial space after myocardial infarction, whereas markers of cardiomyocyte hypertrophy (left ventricular weights and prepro-atrial natriuretic factor [ANF] expression) were not affected. Chronic ACE inhibition reduced collagen deposition and cardiomyocyte hypertrophy after myocardial infarction. The inhibitory action of ACE inhibition on interstitial collagen was partially reversed by B2 kinin receptor blockade. However, B2 kinin receptor blockade did not attenuate the effects of ACE inhibition on cardiomyocyte hypertrophy. In conclusion, kinins inhibit the interstitial accumulation of collagen, but do not modulate cardiomyocyte hypertrophy after myocardial infarction. Kinins contribute to the reduction of myocardial collagen accumulation by ACE inhibition, but the effects of ACE inhibition on cardiomyocyte hypertrophy are related to reduced generation of angiotensin II^52^.

The effect of icatibant (HOE-140), a selective bradykinin receptor antagonist on myocardial IRI, was studied in open-chest barbiturate anaesthetized cats. The left anterior descending coronary artery was occluded for 15 min, followed by 60 min of reperfusion. Saline or icatibant (200 µg/kg) was administered intravenously slowly over 2 and 5 min before reperfusion. In the saline-treated group, MIRI was evidenced by depressed mean arterial pressure (MAP), depressed peak positive and negative dP/dt and elevated left ventricular end-diastolic pressure and enhanced oxidative stress [elevated plasma thiobarbituric acid reactive substances (TBARS; a marker for lipid peroxidation), depressed myocardial GSH (reduced glutathione), SOD, catalase] and depletion of ATP along with rise in plasma creatine phosphokinase. Administration of icatibant resulted in complete hemodynamic recovery together with repletion of ATP and reduction in plasma TBARS without any significant change in myocardial SOD, catalase, and GSH. The results of the present study suggested a protective role of icatibant in myocardial IRI^53^.

Ischemic preconditioning and ranolazine have exhibited cardioprotective effects by reducing infarct size, LDH, CK-MB, and troponin I levels in cardiac injury induced by ischemia-reperfusion. The mechanical functions of cardiac performances like LVDP, dP/dT_max_, and dP/dT_min_ also significantly improved (*p* < 0.05). Previous literature suggests that ischemic preconditioning achieves cardioprotection through pathways involving nitric oxide, adenosine, bradykinin, and ATP-dependent K^+^ channels. Our research aimed to investigate whether ranolazine also engages these pathways to confer cardioprotection.

Our study provides evidence supporting the involvement of nitric oxide, adenosine, bradykinin, and ATP-dependent K^+^ channels in the cardioprotective mechanism of ranolazine. This conclusion is drawn from the observation that inhibitors of these pathways, such as L-NAME and aminoguanidine, nitric oxide inhibitors, abolish the cardioprotective effects of ranolazine. Theophylline and aminophylline adenosine inhibitors abolish the cardioprotective effects of ranolazine. 5-hydroxydecanoate and glimepiride abolish the cardioprotective effects of ranolazine. Conversely, icatibant (bradykinin B2 receptor antagonist, HOE-140) and bromelain (kinins-bradykinin-downregulator), which decrease bradykinin levels, also abolish the cardioprotective effects of ranolazine.

Furthermore, our findings revealed that ranolazine + inhibitors of nitric oxide, adenosine, bradykinin, and ATP-dependent K^+^ channels, such as L-NAME, aminoguanidine, theophylline, aminophylline, icatibant, bromelain, 5-hydroxydecanoate, and glimepiride, leads to an increase in infarct size, LDH, CK-MB, and troponin I levels. The mechanical functions of cardiac performances like LVDP, dP/dT_max_ and dP/dT_min_ also significantly decreases as compared to the ranolazine alone treatment group (*p* < 0.05).

Our research findings underscore the role of nitric oxide, adenosine, bradykinin, and ATP-dependent K^+^ channels in mediating the cardioprotective effects of ranolazine, akin to ischemic preconditioning. Consequently, ranolazine could serve as a pharmacological alternative to surgical ischemic preconditioning before interventional procedures such as coronary artery bypass graft surgery and heart transplantation, offering improved patient compliance by mediating nitric oxide and ATP-dependent K^+^ channels.

## Conclusion

In the ranolazine treated groups administered with inhibitors targeting nitric oxide (L-NAME and aminoguanidine), adenosine inhibitors (theophylline and aminophylline), bradykinin inhibitors (icatibant and bromelain), and ATP-dependent K + channels (5-hydroxydecanoate and glimepiride), there was a notable increase in myocardial infarction percentage, LDH, CK-MB, and troponin I levels. These inhibitors also depress mechanical functions of cardiac performances like LVDP, dP/dT_max_, and dP/dT_min_. Hence, nitric oxide, adenosine, bradykinin release and activation of ATP-dependent K + channels are involved in ranolazine-induced cardioprotection.

In summary, our study findings, combined with existing literature, support the ranolazine involvement of nitric oxide, adenosine, bradykinin, and ATP-dependent K + channels act as secondary messengers in signaling pathways that contribute to the cardioprotective effects of ranolazine, similar to ischemic preconditioning. However, like ischemic preconditioning mediators, ranolazine’s secondary messengers’ involvement still remain and need further research. Therefore, ranolazine could potentially replace surgical ischemic preconditioning and be utilized in ischemic heart disease and before interventional procedures such as coronary artery bypass graft surgery and heart transplantation, offering improved patient compliance during prolonged ischemic periods mediated through signaling nitric oxide, adenosine, bradykinin and ATP-dependent K + channels as secondary messengers.

## Data Availability

Data sharing is not applicable to this article as no new data were created or analyzed in this study.
